# Exploring human resource management in the top five global hospitals: a comparative study

**DOI:** 10.3389/fpubh.2023.1307823

**Published:** 2024-01-05

**Authors:** Xingyou Wang, Richard Szewei Wang, Xiaoping Qin, Yu-Ni Huang, Herng-Chia Chiu, Bing-Long Wang

**Affiliations:** ^1^Fangcao Community Healthcare Center of Chengdu High-Tech Zone, Chengdu, China; ^2^Tsinghua-Berkeley Shenzhen Institute, Tsinghua University, Shenzhen, China; ^3^School of Health Policy and Management, Chinese Academy of Medical Sciences and Peking Union Medical College, Beijing, China; ^4^College of Medical and Health Science, Asia University, Taichung, Taiwan; ^5^Institute for Hospital Management, Tsinghua University, Shenzhen, China; ^6^Bloomberg School of Public Health, Johns Hopkins University, Baltimore, MD, United States

**Keywords:** world’s top hospitals, hospital management, human resource management, organizational culture, value

## Abstract

**Background:**

The pivotal role of Human Resource Management (HRM) in hospital administration has been acknowledged in research, yet the examination of HRM practices in the world’s premier hospitals has been scant.

**Objective:**

This study explored how the world’s leading hospitals attain operational efficiency by optimizing human resource allocation and melding development strategies into their HRM frameworks. A comparative analysis of the HRM frameworks in the top five global hospitals was undertaken to offer a reference model for other hospitals.

**Methods:**

This research offers a comparative exploration of the HRM frameworks utilized by the top five hospitals globally, underscoring both shared and distinct elements. Using a multi-case study methodology, the research scrutinized each hospital’s HRM framework across six modules, drawing literature from publicly accessible sources, including websites, annual reports, and pertinent English-language scholarly literature from platforms such as Google Scholar, PubMed, Medline, and Web of Science.

**Results:**

The analyzed hospitals exhibited inconsistent HRM frameworks, yet all manifested potent organizational cultural attributes and maintained robust employee training and welfare policies. The design of the HR systems was strategically aligned with the hospitals’ objectives, and the study established that maintaining a sustainable talent system is pivotal to achieving hospital excellence.

**Conclusion:**

The HRM frameworks of the five analyzed hospitals align with their developmental strategies and exhibit unique organizational cultural attributes. All five hospitals heavily prioritize aligning employee development with overall hospital growth and place a spotlight on fostering a healthy working environment and nurturing employees’ sense of achievement. While compensation is a notable performance influencer, it is not rigorously tied to workload in these hospitals, with employees receiving mid-to-upper industry-range compensation. Performance assessment criteria focus on job quality and aligning employee actions with organizational values. Comprehensive welfare and protection are afforded to employees across all five hospitals.

## 1 Introduction

Human Resource Management (HRM) navigates through the meticulous planning and rational allocation of human resources, aligning with organizational development strategy by embarking upon a suite of processes like recruiting, training, assignment, evaluation, incentives, and adjustments to maximize personnel value (1, 2). HRM in hospitals emerges as a dynamic key element in the operation and evolution of the facility. Its existence and proficient utilization activate resources effectively, propelling hospitals toward organizational objectives and enhancing healthcare service delivery. Hence, a scientifically sound and efficient HRM becomes indispensable for hospitals to augment their core competitiveness and realize high-quality development (3).

Hospitals, as fundamental entities in the healthcare system, shoulder the vital responsibility of delivering clinical medical services, becoming an essential protective layer for public health (4). The HRM in hospitals, in contrast to other sectors, portrays distinct disparities owing to the specialized service content, clientele, specialized labor divisions, and continuous operation, elevating labor costs and placing specific demands on human resource allocation (5, 6). The heterogeneous and unforeseeable nature of healthcare services also presents notable challenges to internal work process standardization. Thus, maximizing work efficiency through optimal human resource allocation is a vital issue in contemporary hospital management.

The “World’s Best Hospitals 2023” has spotlighted top healthcare institutions globally, with the foremost five being the Mayo Clinic (MC), Cleveland Clinic (CC), Massachusetts General Hospital (MGH), The Johns Hopkins Hospital (JHH), and Toronto General - University Health Network (UHN) (7). Exceptional HRM practices are imperative for delivering top-tier healthcare services and achieving remarkable research output. With a pervasive demand for talent in all hospital positions, adopting apt HRM models and strategies to attract, recruit, train, and retain skilled individuals becomes paramount (3, 8–10).

Despite the availability of individual case studies on HRM in some of the world’s elite hospitals, there prevails a void in comparative research on hospital HRM models (11–14). This study thoroughly analyzes HRM within the aforementioned medical institutions, identifying pertinent takeaways and extracting insightful models of experience. The objective is to offer a referential framework for other hospitals to enhance their HRM models and strategies.”

## 2 Theoretical foundation

**Figure 1:**
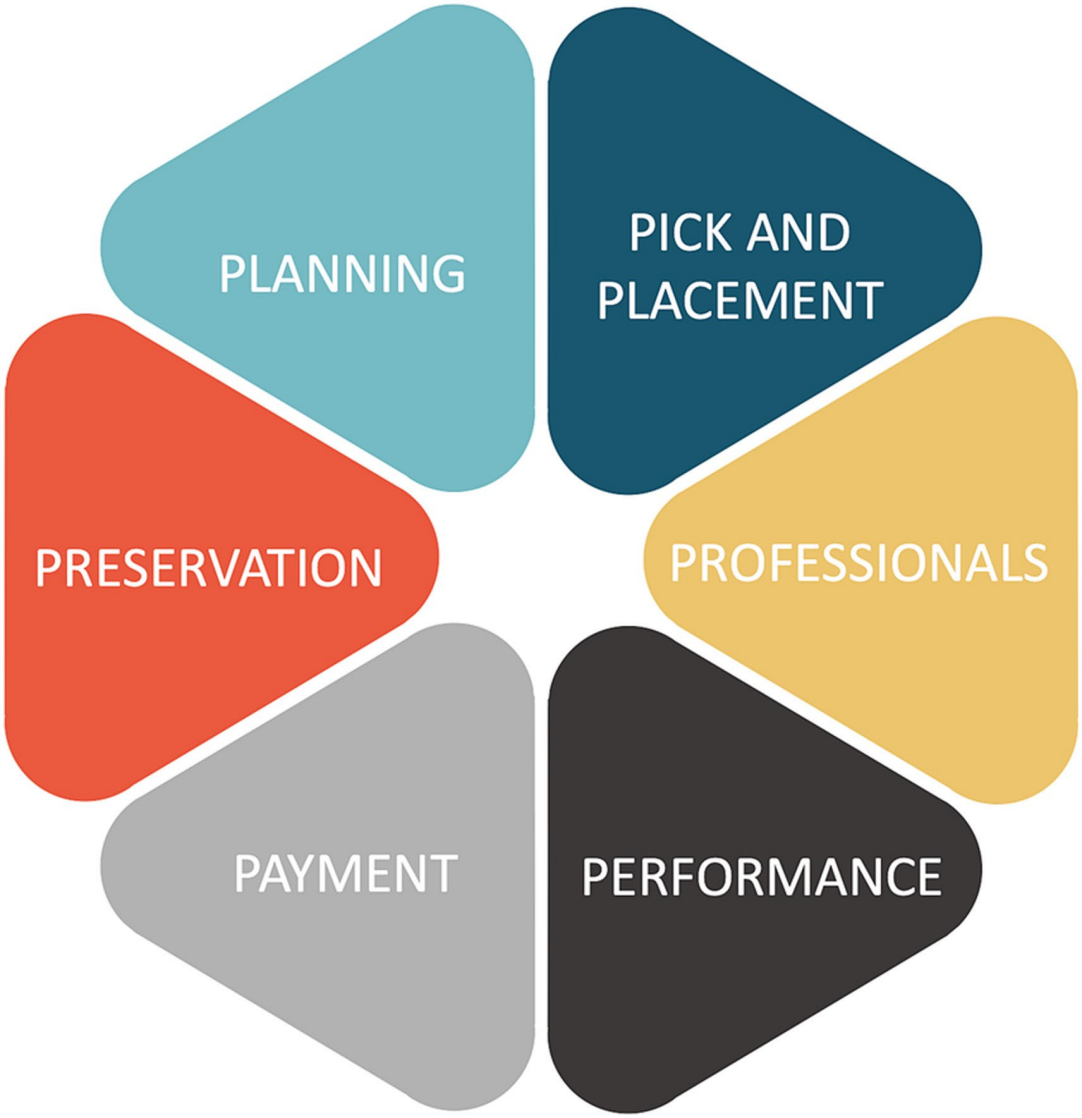
Six modules of human resources management.

The core components of human resources management encompass planning, pick and placement, professionals, performance, payment and preservation (15). This study focuses on a comparative analysis of hospital human resources management across six modules, which are depicted in Figure 1.

Human resource ‘planning’ encompasses the meticulous forecasting of human resource demands and supplies essential for realizing the long-term developmental goals of hospitals (15). The ‘Pick and Placement’ management involves scientifically and judiciously attracting, choosing, and employing individuals in alignment with job prerequisites, ensuring the optimal person is appointed to the correct role at the opportune moment. The concept of ‘professionals’ entails the employer taking accountability for initial job training and ongoing employee development to acquaint them with their role responsibilities, specific task contents, and skill prerequisites while also consistently nurturing them in accordance with the hospital’s career advancement pathways and extensive development plans. ‘Performance’ management encapsulates the thorough monitoring, analysis, and evaluation of the work processes and outcomes of either departments or individual employees. It strives to refine employee behavior and operational processes to pursue the hospital’ s enduring objectives. ‘Payment’ management incorporates both financial and non-financial rewards and emerges as a pivotal component in achieving incentive and performance management goals. ‘Preservation’ management predominantly engages with employee performance assessment, employment, and welfare management. Also, it encompasses the crafting of team building within agencies and the generation of organizational culture and ambiance.

## 3 Methods

In this study, the foremost five hospitals were chosen from ‘World’s Best Hospitals 2023’ (7). The ranking encompasses an extensive list of over 2,300 hospitals across 28 countries and regions, with scoring derived from data involving medical expertise, patient contentment, hospital quality indicators, and patient outcomes.

The primary research methodology employed in this study adhered to a multi-case comparative approach. Engaging in multiple-case research, which involves the comparative analysis of two or more cases under the stewardship of theoretical sampling principles, aims to pinpoint similarities and disparities among the cases under scrutiny and formulate theory (16). When contrasted with single-case research, the multi-case method can more precisely delineate different constructs and their interrelations, pinpointing accurate definitions and appropriate levels of construct abstraction. This approach lays a more robust foundation for theoretical construction and fosters the generation of theories with broader applicability (16, 17). The data for this research was sourced from publicly available information on the five hospitals, including official websites, annual reports, and pertinent English academic literature on platforms like Google Scholar, PubMed, Medline, and Web of Science (WOS). It is pivotal to note that the research materials were derived from public domains and did not engage in any biological research involving humans or animals.

## 4 Results

The five hospitals are all non-profit organizations with a history of over 100 years. MC, CC, MGH, and JHH are in the United States, while UHN is in Canada. Four hospitals are private institutions except for UHN. The basic information of the five hospitals can be found in Table 1.

**Table 1:** Basic information of the five hospitals.

Hospital	MC	CC	MGH	UHN	JHH
Ranking 2023	1	2	3	4	5
Headquarters	Rochester, Minnesota, United States	Cleveland, Ohio, United States	Boston, Massachusetts, United States	Toronto, Ontario, Canada	Baltimore, Maryland, United States
Since	1883	1921	1811	1829	1889
Care System	Private	Private	Private	Public	Private
Beds	1,243	-	927	-	-
Number of Employees	73,600	-	23,173	-	10,400
Beds-to-Employee Ratio	0.017	-	0.04	-	-
Annual Outpatient Cases	Over 1.4 million	-	1,440,548	-	-
Campuses & Branches	3 & 2	5 & 4	1 & 0	3 & 2	1 & 0
University/Affiliated	Mayo clinic school of medicine	Cleveland clinic lerner college of medicine	Harvard university	University of Toronto	Johns Hopkins university

### 4.1 Planning

As shown in Table 2, each hospital has unique characteristics in human resources planning. At MC, employee cost analysis is performed and information systems are used for business prediction to clarify the hospital development scale and corresponding personnel needs. Remote full-time positions are created based on job content to overcome the spatial limitations of the workplace, improve work efficiency and output quality, and incorporate human resources planning indicators into hospital development strategies. At CC, human resources planning is independently borne by the medical group, with dedicated management departments responsible for personnel recruitment, cost control, and hospital profit and loss. MGH has “The Massachusetts General Physicians Organization” serving as an independent organization responsible for the appointment and removal of medical personnel and the development and modification of related rules and regulations. UHN formulates human resources strategies and job responsibilities based on the hospital’s development vision and goals, with a highly-developed employee welfare system that encourages employee participation in hospital management practices. At JHH, a data-driven approach is adopted for human resources planning and analysis, using personnel analysis, labor analysis, or talent analysis as the basis for personnel management to improve decision-making.

**Table 2:** Characteristics of human resources planning in five hospitals.

Hospital	Characteristics
MC	Human resources planning indicators are datamined and integrated into the overall hospital strategy
CC	The medical group is independently responsible for the planning
MGH	Managed by the Massachusetts General Hospital Physicians Organization as an independent institution
UHN	Developed in line with the hospital’s vision and goals
JHH	Adoption of a data-driven approach to human resources planning.

### 4.2 Pick and placement

Table 3 presents the recruitment and staffing features of each hospital. MC follows the “values first” principle and emphasizes the alignment of values by selecting candidates who embrace Mayo’s value “The needs of the patient come first” through at least two rounds of interviews. Candidates are selected through at least two rounds of interviews and are regularized after an initial probationary period and an in-depth probationary period of about 3 years. A variety of corporate culture training programs are also in place to emphasize diversity in talent selection and to increase the percentage of minorities among employees.

**Table 3:** Characteristics of human resources pick and placement in five hospitals.

Hospital	Characteristics
MC	1. Equal emphasis on professional skills and value consistency; 2. Focus on the diversity of backgrounds of talents
CC	Management positions are filled by skilled professionals with medical backgrounds to minimize management decision-making errors
MGH	The Personnel Specialist assists section managers in the implementation of specific human resources management tasks
UHN	Use of the Internet, partnerships with colleges and universities, and student recruitment programs
JHH	Recruitment and onboarding of employees managed by the HR team

To avoid conflicts between management decisions and the best medical decisions, management positions at CC are filled by technical professionals with medical backgrounds. When recruiting managers, candidates are screened by management and are required to choose between management and clinical career paths, and are recognized by management before entering management. From the management to the medical staff, all of them have specialized medical knowledge. With a clear division of labor in the team, doctors and nursing staff can be exempted from involvement in matters other than therapeutic care, reducing the risk of medical corruption (18).

The recruitment and staffing of MGH is the responsibility of the leaders within the Massachusetts General Hospital Physicians Organization (MGHPO), in conjunction with the hospital president. Departments and physicians are supported by secretaries for day-to-day management to increase efficiency (19). Each department has an administrative director with a professional management background in addition to the department chair. The administrative director is responsible for the development strategy, finance, personnel, and operation of the department. In terms of human resource management positions, the personnel commissioner model and the departmental responsibility system are adopted to achieve a high degree of compatibility between human resource management and departmental management.

UHN utilizes various means such as Internet recruitment, university collaborations, student recruitment programs, and other strategies to broaden the applicant pool and widely attract top talent (20). Full-time recruiters are established to address job vacancies, collaborating with educational experts to ensure a talent pipeline and bridging the gap between hospital talent needs and university training/recruitment efforts (21). Its student recruitment program, which includes co-op, summer, internship, and post-graduate programs, involves nine colleges and universities that fulfill the hospital’s need to hire more students to address short-term, high workloads and projects in addition to traditional full-time recruiters. JHH follows a traditional, centralized HRM model, with the Career Services team in the Human Resources Department handling all new hires in a centralized manner.

JHH uses a traditional centralized HRM model, with the Career Services team in the Human Resources Department handling the recruitment and on-boarding of its new employees.

### 4.3 Professionals

The characteristics of training and HRD in each hospital are summarized in Table 4. MC provides various training programs and learning opportunities for all employees, emphasizing the training of technical staff with humanistic content such as leadership and career goal planning. All new employees undergo training on the values of “The needs of the patient come first.” Each healthcare worker is given the opportunity to become a manager in the corresponding department or division, and evaluation indicators are introduced to monitor the results of staff training.

**Table 4:** Characteristics of human resources professionals in five hospitals.

Hospital	Characteristics
MC	1. Provide training opportunities for all employees; 2. Use evaluation indicators to test the results of employee training.
CC	1. Diversified course options, combining online and offline, medical and non-medical educational activities; 2. Training courses in cooperation with external institutions
MGH	1. Each staff member is required to receive training and pass an assessment; 2. Set up a career development center to provide staff with guidance and assistance in all areas according to their needs; 3. Set up a dedicated department to ensure that staff personal growth goals and departmental development goals are harmonized; 4. Arrange for managers to receive leadership training
UHN	1. Establishment of mandatory online learning courses; 2. Structured career paths
JHH	1. Job-related training is conducted by the Office of Human Resource Management Strategic Development; 2. REACH helps employees gain the skills and knowledge needed to
	fill vacant positions

CC places a strong emphasis on personnel training, offering more than 50 courses each year internally, which have been attended by a cumulative total of over 7,000 professionals and technicians. The education and training of medical professionals and technicians is carried out through collaborations with several universities and organizations both domestically and internationally, as well as other medical institutions. For example, continuing medical and non-continuing medical education activities are conducted by the Center for Continuing Education in cooperation with the Institute for Quality and Patient Safety; continuing medical education is conducted through online learning and on-site regular educational activities. Special communication experience courses are offered, leadership training is provided for medical staff who do not have a management background, and a professional management team is set up to assist in administrative work, ensuring that professionals and technicians can remain focused on clinical practice.

MGH regards its staff as the hospital’s greatest asset, has a comprehensive training system, and adheres to the principle that no one can be employed without completing the training program. Its Career Development Center includes the Clinical Career Development Office, the Research Staff Development Office and the Women’s Development Office. It provides daily training and is responsible for the annual Career Development Conference. Through the Annual Career Conference, employees’ personal expectations are aligned with their growth goals and departmental development goals, enabling them to better serve the organization’s common interests while achieving good development in their personal careers. Each department discusses with employees their personal growth goals and departmental development goals to find common ground and make work plans and career plans for the following year. Guidance and assistance are provided to employees according to their needs, and a variety of scholarships and grants are available to help alleviate the financial burden of learning new knowledge and skills, such as the Tuition Assistance Program. Managers need to receive leadership training. A “Management Trainee” program is offered to provide 2 years of training to promising talent. Interns are assigned to different departments for internships and can also join the hospital’s senior operations team to learn and practice.

UHN has separate mandatory e-learning courses for different categories of employees, and designs structured career paths that provide clear opportunities for advancement for individuals with the right skills and experience. A professional development budget is established for course fees, book purchases, and conference planning in the areas of project management and application support. Employees who receive educational development grants are required to submit appropriate training reports for review by all department members. Team-building workshops are held to encourage employees to share their expertise with colleagues (22).

JHH’s Human Resources Department develops training programs based on the needs of the organization and integrates on-the-job training with the organization’s performance goals. The Office of Strategic Workforce Development (SWD) provides advice and assistance to employees in identifying next steps and developing plans to achieve their goals. SWD through the Hopkins Resources and Education for Career Development (REACH) program, provides the JHH system’s SWD provides in-person and online career guidance to individuals and groups through the Hopkins Career Development Resources and Education (REACH) program, which identifies appropriate openings for current employees and provides career development counseling to current employees and community adults and youth, and participates in this effort in partnership with Johns Hopkins University.

### 4.4 Performance

The characteristics of each hospital’s performance management can be seen in Table 5. The value concept of MC runs through the whole process of its human resource management. The purpose of performance appraisal is to help employees improve their competence and is not linked to salary or bonus. The performance appraisal includes three areas: medical care, medical education and medical research (the triple shield). The employee’s annual performance review consists of a self-assessment and a supervisor’s evaluation. The appraisal includes the core values of each position, required professional competencies, achievement of annual goals, and an overall assessment of accomplishments. The results of the performance review are used as a guide for ongoing employee training rather than as a basis for termination (23). Integration of HR assessment and financial performance evaluation with organizational development strategies using HR balanced scorecard, human capital return on investment metrics with non-profit strategy maps. Diversified incentive models such as spiritual rewards (e.g., Karis Quarterly Awards) and academic evaluation systems were used to comprehensively assess healthcare workers’ contributions.

**Table 5:** Characteristics of human resources performance in five hospitals.

Hospital	Characteristics
MC	1. Performance appraisal has nothing to do with workload, and the results of performance appraisal serve as a basis for hospitals to develop skills for their employees; 2. Enhance employee performance through diverse incentive models
CC	1. A comprehensive performance evaluation system; 2. Create special awards to recognize exemplary service and dedication to excellence
MGH	Multiple awards to recognize outstanding employees
UHN	Establishment of special awards to recognize performance in daily work in line with organizational values
JHH	1. use an electronic performance appraisal system; 2. Set up employee appreciation and recognition programs

The most important feature of CC’s human resource management model is its high-frequency and flexible performance appraisal approach. Measures such as the development of implementation processes, the creation of evaluation charts, and performance appraisals ensure the continuous progress of the organization, and CC healthcare workers can plan their work and allocate their time according to their personal wishes to achieve optimal delivery of their work through a high-frequency evaluation cycle once a year. There is Maria and Sam Miller Professional Excellence Award that recognizes exemplary service and dedication to excellence.

MGH has adopted a cultural assessment model for year-end appraisals, with high performance ratios to motivate employees and integrate their values into the organization’s operations, such as the “icare in Action” program that recognizes individuals and teams who have been singled out in patient thank-you letters. The annual Popular Patient Experience Award recognizes extraordinary dedication, and recipients are honored at an annual ceremony hosted by senior executives.

UHN takes a similar approach to performance assessment, such as the “Living Our Values Awards,” which recognizes outstanding employees by the results of living the organization’s values (safety, caring, teamwork, integrity, and stewardship) in their daily work.

JHH uses an online electronic performance management system in conjunction with Johns Hopkins, and human resources departments provide department heads with the tools and resources they need to conduct performance reviews, assisting with departmental payroll and providing them with the tools and resources they need to conduct performance reviews. Tools and resources to assist in departmental salary planning decisions. Employee appreciation programs are also set up to strengthen the positive guiding role of organizational culture.

### 4.5 Payment

The compensation management of each hospital is characterized as follows in Table 6. MC has a pre-determined target annual salary based on job position, with equal pay for equal work, and it does not have a pure salary system based on performance incentives. Compensation is determined based on a combination of an individual’s contribution to the organization and their competence rather than job position. Regular reviews and internal satisfaction surveys are conducted by the Remuneration Officer. Remuneration levels are higher than the industry average (at the 60th percentile). MC has adopted a fixed annual salary system that decouples performance from salary income and uses remuneration, as well as diversified non-material incentives and benefits policies, to provide employees with motivation (24).

**Table 6:** Characteristics of human resources payment in five hospitals.

Hospital	Characteristics
MC	Adoption of a fixed annual salary system to motivate employees with diversified non- material incentives and welfare policies
CC	Healthcare workers’ earnings are not related to bonuses or workload
MGH	Higher salary expenditure as a percentage of total expenditure
UHN	Establishment of a dynamic pay monitoring and adjustment strategy
JHH	Employee salaries are higher than the average income level of peers

CC healthcare workers’ income is not linked to the volume of treatment, and its salary and benefit expenses account for 56% of the center’s operating income, with this high proportion ensuring that healthcare workers’ income remains in the upper middle of the industry within its strict payroll system (25). MGH operates a relatively similar compensation mechanism to CC, with employee salary expenses accounting for approximately 42% of total expenses (26).

UHN utilizes external benchmark for pay adjustments, using benchmark jobs and extracting market salary data at the 50th and 75th percentiles. In-depth research was also conducted on hospitals, leading to the benchmark of pay levels in five hospitals with a dynamic adjustment mechanism in place to ensure that their pay levels were appropriate. Staged salary reviews and adjustments were conducted every 3 months, and market analysis reviews and adjustments were conducted every 6 months to ensure fairness by modifying pay policies for overtime, return to work at any time, and shift work (27). A relatively independent and centralized personnel management model has been adopted at JHH. Employee payroll is accounted for and paid by the Payroll Shared Services Division of the Human Resources Information Processing Center (HRIPC).

### 4.6 Preservation

The characteristics of each hospital’s labor relations and benefits management are summarized in Table 7. MC assesses employee satisfaction through employee surveys and third-party evaluations with the aim of reducing turnover rates through high levels of employee satisfaction. Special attention is paid to highly mobile employee groups, such as registered nurses, and in-depth studies are conducted through focus group interviews and other means to identify key causes and take remedial action. Employees are provided with a comprehensive benefits package including medical, dental, tuition reimbursement, paid leave, and defined benefit pension plans, etc. CC signs one-year term contracts with all employees, including top management, conducts annual reassessments and appointments, conducts annual professional reviews, and adopts an accountable model of contractual appraisal.

**Table 7:** Characteristics of human resources preservation in five hospitals.

Hospital	Characteristics
MC	Ensure high levels of employee satisfaction to improve talent retention rates. Focus on high turnover employee groups, identify the key causes, and take corrective actions. Provide employees with a comprehensive benefits package.
CC	Conduct of annual staff-wide evaluations and annual reassessments and appointments
MGH	1. Free and Confidential Employee Assistance Program to help employees achieve work-life balance; 2. Generous Employee Benefits Package
UHN	Performing Motivational Analysis on Departing Employees for Continuous Improvement
JHH	1. prioritize identifying and retaining talent while fostering a strong sense of organizational identity among employees; 2. implement employee assistance initiatives

MGH operates a free and confidential “Employee Assistance Program” to help employees achieve work-life balance; it is not only a counseling office but also an online community called “myStrength” where employees can find solutions to their problems and interact with other members of the community; and a generous employee benefits package that includes healthcare, retirement benefits, employee wellness program activities, childcare centers, consumer discounts, pet insurance, and transportation subsidies.

UHN conducts surveys for departing employees to explore the reasons for their departure and analyze their motivations, including salary, workload, career opportunities, and professional development (28). JHH has an Applause program for employee recognition, which consists of several components, including service anniversary recognition, peer recognition, manager recognition, and birthday recognition. Similar to MGH, JHH has an “Employee Assistance Program” (JHEAP) to help employees through difficult stages.

## 5 Discussion

As the world’s top hospitals, these five hospitals undoubtedly have some commonalities and differences in human resource management concepts and practices. Examining the similarities and differences while analyzing the potential causes or effects requires consideration of the following six modules of human resource management.

### 5.1 Planning

All five hospitals have integrated the overall strategic direction into their human resources planning. Both the MC in the United States and UHN in Canada have conducted HR planning analyzes based on data indicator tools. Compared with the MC’s analysis system, which is more refined and covers indicators of different dimensions such as employee cost analysis and return on human capital investment indicators, these tools provide sufficient rationales for human resources decision-making and enhance the scientific nature of human resources planning. MC, CC, and MGH will be responsible for the human resources planning functions from the medical institutions, medical groups, and administrative teams that are responsible for the relevant work. This approach enables medical professionals and technicians to focus on the diagnosis and treatment of patient services, while professional managers carry out human resources planning and daily management. Human resources planning needs to be integrated with the organization’s development strategy to maintain consistency in management implementation. The concept of strategy first is adhered to in manpower planning, and the medium- and long-term development plan of the institution is used as a starting point to determine the appropriate organizational structure and team model for human resources planning.

### 5.2 Pick and placement

In terms of recruitment and staffing, the MC ensures that employees who share the organization’s values are selected and retained by means of a values-based recruitment philosophy and a long recruitment, training, and probationary period designed to foster the organization’s long-term growth and vision. MC departments are managed through a “partner leadership” system, in which physician specialists and professional managers work together to make management decisions. The effective functioning of this model depends on the degree of coordination between the physicians and managers, who need to focus on patient care, and the managers who need to be accountable to shareholders and ensure that the organization operates efficiently and appropriately in terms of revenue. On the other hand, UHN achieves an adequate supply of quality human resources through programs such as the Student Recruitment Program, which is closely related to its status as a teaching hospital of the University of Toronto. The departments and staffing at the five hospitals are quite different. In general, hospitals will tailor their recruitment strategies and employee retention measures according to their specific organizational culture, strategic goals, and resource allocation.

MHG’s “dual director” system is similar to the MC’s “dual-track” mechanism, with the department director responsible for medical, academic, and other professional areas, and the administrative director responsible for departmental development strategy, finance, personnel, operations, and other work. This approach helps to avoid the department director being trapped in complex administrative affairs and unable to focus on business work, while also assisting in realizing the specialization and refinement of management. CC uses personnel with medical backgrounds to ensure that decisions do not deviate from specialized medical knowledge. At the same time, through clear division of labor in the team, medical staff can focus on clinical diagnosis and treatment and nursing care, which is similar to Mayo’s practice. JHH has a relatively centralized human resources management staffing structure, with a vice president in charge of different areas of the organization, including a director of pensions, a human resources consultant, a director of human resources development and training, a director of recruiting, and a director of compensation and benefits. MGH has a management specialist in charge of all types of human resources management, enhancing the ease of interface with human resources services for departments and employees.

The five institutions in this study do not have the same organizational structure and team model, but they have all achieved outstanding development results, demonstrating that both traditional departmental settings and innovative hospital structures can achieve quality development, but must be accompanied by appropriate management models. Human resource planning based on the organization’s strategy can maintain consistency in management at the same stage and avoid rotation in key positions.

### 5.3 Professionals

In terms of staff training and human resources development, all five hospitals attach great importance to the continuing education and diversified training of their staff. The content of the training is unique, but all of them involve the development of comprehensive qualities in various aspects rather than being limited to clinical skills. Many hospitals also emphasize leadership training programs: MC focuses on improving the leadership, goal-planning, and other personal qualities of professional and technical staff; CC offers various leadership programs for clinical staff; MGH focuses on training for managers; JHH implements leadership development through courses, tasks, and other multidimensional means. Leadership is a quality that should be possessed by all types of workers and contributes to superior performance and operational outcomes in hospital organizations.

In addition, the curricula of the five hospitals are unique: Mayo Clinic emphasizes training on values, Cleveland Clinic offers communication experience courses and has built a comprehensive online training system, MGH customizes training programs for individual needs, UHN encourages peer-experience-sharing-oriented learning through a series of seminars, and JHH customizes its training programs from the perspective of talent identification and development plan development. It is worth noting that each hospital places considerable emphasis on the career planning and development of individual employees. Mayo Clinic uses an evaluation and monitoring system to track employee development, MGH helps employees adjust their plans through an annual career development meeting system, UHN has a separate career development budget to provide career path options for employees, and JHH helps employees formulate their career development plans through a comprehensive assessment. It can be seen that the development of an organization cannot be separated from the development of each individual; the two are complementary, and an excellent organization will do its best to help rather than limit the development of individual members.

Prioritize the development of individuals with diverse expertise, fostering administrative teams that function effectively in both managerial and clinical capacities, while also elevating the professional competence of the leadership knowledge of clinical medical professionals. The allocation of human resources in hospital management positions directly affects the overall level and efficiency of hospital management. Because hospital management is in the interdisciplinary field of medicine and management, the output of relevant professionals is relatively small, many hospitals are still lacking in specialized management personnel, mostly doctors and nursing staff directly, resulting in the de-professionalization of hospital management, and this kind of sloppy management brings about the disorder of hospital governance. Therefore, the optimal allocation of human resources for hospital management positions can be achieved by introducing and training professional hospital managers and improving the management literacy of clinical workers.

### 5.4 Performance

In terms of performance management, MC’s performance management model is different from the other four hospitals and has its own system. Firstly, it divides performance appraisal into five levels from macro to micro, i.e., strategic performance - corporate performance - the triple shield performance (medical care, medical education and medical research) - the performance of various medical services - employee performance, thus realizing the organic link between individual employee performance and the overall organizational performance. Secondly, MC combines individual performance appraisal with performance appraisal of overall HR work through various quantitative indicators and a values-based multidimensional appraisal system, the results of which are used for feedback to individual employees and hospital management as follow-up guidance.

It is worth noting that all five hospitals have established non-material rewards as an important part of employee performance evaluation. For example, MC has the Karis Quarterly Award, CC has the Maria and Sam Miller Professional Excellence Award, MGH has the Annual Patient Experience Award, UHN has the Living Our Values Award, and JHH has the Employee Appreciation and Recognition Program. Different types of non-material rewards are established to recognize the outstanding contributions of employees from different perspectives and to give excellent employees a sense of spiritual satisfaction so that they can execute their quality beliefs more firmly. At the same time, the establishment of awards targeted at teamwork and evaluation by others is also conducive to enhancing the cohesion among employees.

With cultural guidance as the core complemented by the use of scientific management tools, emphasis is placed on the shaping of corporate culture. The five hospitals studied all have distinctive corporate cultures and values, and their cultural infiltration is reflected in all aspects of human resource management. There is even a lack of performance evaluation systems that use cultural identity as an assessment indicator. The advantage of cultural guidance is to ensure consistency of internal values, ease in implementing management decisions, and further ensure the quality of medical services through consistency of values and teamwork harmony. However, the formation of corporate culture takes a long time and places high demands on core managers, making it difficult to form in a short period of time and easy to assess. Therefore, it is necessary to introduce the use of scientific management tools. Management tools serve as an objective evaluation mechanism combined with cultural guidance. Before the formation of a stable and positive corporate culture, the system serves as an objective assessment mechanism; in a stable and positive corporate atmosphere, it can also be used as a management supplement, ensuring fairness and objectivity of the assessment while reducing the subjectivity of the assessment caused by changes in management personnel.

### 5.5 Payment

In terms of compensation, the fixed compensation of MC and CC lacks the incentive effect of “more work, more pay,” but it has not caused slackness in work and lower quality of work. This is because the attractiveness of the clinic’s own work platform and the competitive level of salary offered enable employees to obtain reasonable returns. On the other hand, the two clinics also use other means to make their employees unwilling or afraid to relax their work requirements. MC adopts more mental incentives and a comprehensive welfare program, while CC uses contractual appraisals to make employees take their work seriously. The pay adjustment system of UHN follows a cyclical approach to ensure that real-time dynamics allow employees to receive pay in line with the market level, thus ensuring the scientific nature of pay setting, which neither over-motivates nor avoids the lack of motivation caused by under-motivation.

To emphasize humanistic management and non-economic rewards, upholding the concept of humanistic care while focusing the needs of employees. The five healthcare organizations in this study view employee welfare as an important part of human resource management. Not only is career development an important element, but there are also corresponding welfare measures and protection mechanisms for their personal and family members’ education, health, and economic status. As a talent-intensive industry, healthcare employees are highly educated and value personal development more than financial compensation. The five organizations have invested significant human and material resources in building a high-quality employee welfare system. For example, the Employee Assistance Program of MGH was designed to help employees balance work and life, creating a good environment for employees to be able to devote themselves to their work. Employees can seek help from the program office for any problems they may have. There are strong professional barriers in the medical services industry, with both the organization and patients seeking the purity of medical services. High-quality employee services are provided to employees to solve their concerns, while ensuring the simplicity of medical services and avoiding the impact on patient safety and treatment effects due to additional performance issues.

### 5.6 Preservation

Employee satisfaction and turnover are indicators that several hospitals focus on in different ways. Mayo Clinic comprehensively understands employee satisfaction through surveys, staff meetings, third-party observations and other means, and analyzes the motivation for leaving through focus group interviews and other research methods. Toronto General Hospital conducts a similar analysis of reasons for leaving and uses it to develop talent retention strategies. Loss of professional talents is a bottleneck problem facing all regions and types of hospitals, and due to the long cycle and high cost of training medical professionals, the loss of talents to hospitals is extremely serious, so talent retention is an important issue of concern to the world’s leading hospitals.

These hospitals place great emphasis on employee welfare and have designed detailed welfare programs that consider various aspects of employees’ lives and work, enabling them to work with peace of mind. Although these welfare programs may seem to increase the cost of hospitals, they are significant in terms of improving employee efficiency and sense of belonging. Soft benefits not only compensate for limitations in salary but also reflect the “people-oriented” value concept, making employees feel the hospital organization’s care and support for individuals, and thus more motivated to perform their work. In terms of labor relations management, CC adopts a yearly contract evaluation model and renewal of employment on an annual basis, which avoids to some extent the loss of a sense of crisis and stability brought about by long-term or open-ended contracts, and necessitates all types of employees to maintain a rigorous and conscientious attitude toward their work.

### 5.7 Strengths and limitations

This study provides an in-depth analysis of HRM practices within the specified medical institutions, discerning key insights and deriving valuable models from their experiences. It proposes a framework as a reference for other hospitals looking to bolster their HRM models and strategies. However, it is essential to acknowledge certain limitations within this study. One such limitation pertains to the representativeness and generalizability of the sample cases, which necessitates further enhancement. The applicability of these cases to HRM practices in hospitals across diverse nations and systems also requires additional exploration.

Furthermore, the literature review is constrained by information sources, making it challenging to accurately and comprehensively reconstruct human resource management practices in various hospitals compared to field research and alternative research methodologies. The content differences in the public data of these hospitals make it challenging to acquire more comprehensive details about their human resource management systems and the actual implementation of these systems. Therefore, there could be potential discrepancies resulting from variations in the disclosure of information across healthcare organizations. More comprehensive information will be obtained through field research or in-depth interviews for comparative studies in the future. It is the hope that future research endeavors will see more researchers and teams contributing to empirical research outcomes in the global field of hospital human resource management. This would further refine and augment resources for hospitals worldwide, aiding in improving their HRM endeavors and realizing high-quality development.

However, the overall ranking of the journals is based on international recommendations from peers in different countries and incorporates the hospital scores. Therefore, the top five hospitals in the ranking are comparable.

## 6 Conclusion

The study provides an in-depth analysis of Human Resource Management (HRM) practices within five top-ranking global hospitals, unveiling both universal and unique strategies to pave the way for innovative HRM in other hospitals. The insights underscore the critical alignment of human resources planning with an organization’s developmental strategy, ensuring management consistency and strategic personnel placement. Success has been witnessed through both traditional and innovative organizational structures when paired with apt management models. A distinctive corporate culture and value system have been pivotal, although their formation demands considerable time and managerial input, highlighting the need for scientific management tools for objective evaluation. A significant focus is placed on nurturing multidisciplinary talents and creating cohesive administrative teams, addressing the current deficit of specialized management personnel in many hospitals. The analysis aims to provide a blueprint for improving HRM strategies in healthcare institutions worldwide, considering the divergences and similarities in HRM practices across eminent hospitals. Future investigations should explore how specific information technologies have been used to promote the effectiveness and efficiency of human resource management in large hospitals.

## Data availability statement

The original contributions presented in the study are included in the article/Supplementary material, further inquiries can be directed to the corresponding authors.

## Author contributions

XW: Writing – original draft, Data curation, Methodology. RW: Conceptualization, Data curation, Writing – original draft. XQ: Writing – review & editing, Data curation, Investigation. Y-NH: Data curation, Writing – review & editing. H-CC: Writing – review & editing, Conceptualization, Methodology, Project administration, Supervision. B-LW: Conceptualization, Methodology, Project administration, Supervision, Writing – review & editing, Funding acquisition.
