# Supplementary material for: Exploring human resource management in the top five global hospitals: a comparative study
Source: Front Public Health. 2024 Jan 5;11:1307823. doi: 10.3389/fpubh.2023.1307823 (PMC10799338; doi:10.3389/fpubh.2023.1307823)
Supplement: Supplementary file 1 [file Table_1.DOCX]

Supplementary Material

# Supplementary Figures and Tables

## Supplementary Figures

**Supplementary Figure 1.** Six Modules of Human Resources Management

## Supplementary Tables

**Supplementary Table 1.** Basic Information of the Five Hospitals

| **Hospital** | **MC** | **CC** | **MGH** | **UHN** | **JHH** |
| --- | --- | --- | --- | --- | --- |
| Ranking 2023 | 1 | 2 | 3 | 4 | 5 |
| Headquarters | Rochester,  Minnesota, USA | Cleveland, Ohio, USA | Boston,  Massachusetts, USA | Toronto, Ontario, Canada | Baltimore, Maryland, USA |
| Since | 1883 | 1921 | 1811 | 1829 | 1889 |
| Care System | Private | Private | Private | Public | Private |
| Beds | 1243 | - | 927 | - | - |
| Number of Employees | 73600 | - | 23173 | - | 10400 |
| Beds-to-Employee Ratio | 0.017 | - | 0.04 | - | - |
| Annual Outpatient Cases | Over 1.4 million | - | 1440548 | - | - |
| Campuses & Branches | 3 & 2 | 5 & 4 | 1 & 0 | 3 & 2 | 1 & 0 |
| University/Affiliated | Mayo Clinic School of Medicine | Cleveland Clinic Lerner College of Medicine | Harvard University | University of Toronto | Johns Hopkins University |

**Supplementary Table 2.** Characteristics of Human Resources Planning in Five Hospitals

| **Hospital** | **Characteristics** |
| --- | --- |
| MC | Human resources planning indicators are datamined and integrated into the overall hospital strategy |
| CC | The medical group is independently responsible for the planning |
| MGH | Managed by the Massachusetts General Hospital Physicians Organization as an independent institution |
| UHN | Developed in line with the hospital's vision and goals |
| JHH | Adoption of a data-driven approach to human resources planning. |

**Supplementary Table 3.** Characteristics of Human Resources Pick and Placement in Five Hospitals

| **Hospital** | **Characteristics** |
| --- | --- |
| MC | 1.Equal emphasis on professional skills and value consistency; 2. Focus on the diversity of backgrounds of talents |
| CC | Management positions are ﬁlled by skilled professionals with medical backgrounds to minimize management decision-making errors |
| MGH | The Personnel Specialist assists section managers in the implementation of speciﬁc human resources management tasks |
| UHN | Use of the Internet, partnerships with colleges and universities, and student recruitment programs |
| JHH | Recruitment and onboarding of employees managed by the HR team |

**Supplementary Table 4.** Characteristics of Human Resources Professionals in Five Hospitals

| **Hospital** | **Characteristics** |
| --- | --- |
| MC | 1.Provide training opportunities for all employees; 2. Use evaluation indicators to test the results of employee training. |
| CC | 1.Diversiﬁed course options, combining online and ofﬂine, medical and non-medical educational activities; 2. Training courses in cooperation with external institutions |
| MGH | 1.Each staff member is required to receive training and pass an assessment; 2. Set up a career development center to provide staff with guidance and assistance in all areas according to their needs; 3.Set up a dedicated department to ensure that staff personal growth goals and departmental development goals are harmonized; 4.Arrange for managers to receive leadership training |
| UHN | 1.Establishment of mandatory online learning courses; 2. Structured career paths |
| JHH | 1.Job-related training is conducted by the Ofﬁce of Human Resource Management Strategic Development; 2. REACH helps employees gain the skills and knowledge needed to |
|  | ﬁll vacant positions |

**Supplementary Table 5.** Characteristics of Human Resources Performance in Five Hospitals

| **Hospital** | **Characteristics** |
| --- | --- |
| MC | 1. Performance appraisal has nothing to do with workload, and the results of performance appraisal serve as a basis for hospitals to develop skills for their employees; 2. Enhance employee performance through diverse incentive models |
| CC | 1.A comprehensive performance evaluation system; 2. Create special awards to recognize exemplary service and dedication to excellence |
| MGH | Multiple awards to recognize outstanding employees |
| UHN | Establishment of special awards to recognize performance in daily work in line with organizational values |
| JHH | 1. use an electronic performance appraisal system; 2.Set up employee appreciation and recognition programs |

**Supplementary Table 6.** Characteristics of Human Resources Payment in Five Hospitals

| **Hospital** | **Characteristics** |
| --- | --- |
| MC | Adoption of a ﬁxed annual salary system to motivate employees with diversiﬁed non- material incentives and welfare policies |
| CC | Healthcare workers' earnings are not related to bonuses or workload |
| MGH | Higher salary expenditure as a percentage of total expenditure |
| UHN | Establishment of a dynamic pay monitoring and adjustment strategy |
| JHH | Employee salaries are higher than the average income level of peers |

**Supplementary Table 7.** Characteristics of Human Resources Preservation in Five Hospitals

| **Hospital** | **Characteristics** |
| --- | --- |
| MC | Ensure high levels of employee satisfaction to improve talent retention rates. Focus on high turnover employee groups, identify the key causes, and take corrective actions. Provide employees with a comprehensive beneﬁts package. |
| CC | Conduct of annual staff-wide evaluations and annual reassessments and appointments |
| MGH | 1.Free and Conﬁdential Employee Assistance Program to help employees achieve work-life balance; 2.Generous Employee Beneﬁts Package |
| UHN | Performing Motivational Analysis on Departing Employees for Continuous Improvement |
| JHH | 1. prioritize identifying and retaining talent while fostering a strong sense of organizational identity among employees; 2. implement employee assistance initiatives |
